# Best imaging signs identified by radiomics could outperform the model: application to differentiating lung carcinoid tumors from atypical hamartomas

**DOI:** 10.1186/s13244-023-01484-9

**Published:** 2023-09-19

**Authors:** Paul Habert, Antoine Decoux, Lilia Chermati, Laure Gibault, Pascal Thomas, Arthur Varoquaux, Françoise Le Pimpec-Barthes, Armelle Arnoux, Loïc Juquel, Kathia Chaumoitre, Stéphane Garcia, Jean-Yves Gaubert, Loïc Duron, Laure Fournier

**Affiliations:** 1 Imaging Department, Hopital Nord, APHM, Aix Marseille University, Marseille, France; 2 LIIE, Aix Marseille Univ, Marseille, France; 3 PARCC UMRS 970, INSERM, Université Paris Cité, Paris, France; 4 Department of Pathology, Hôpital Européen Georges Pompidou, Assistance, Publique Hôpitaux de Paris, Paris, France; 5 Service de Chirurgie Thoracique et Transplantation Pulmonaire, Hôpital Nord, Chemin des Bourrely, Aix Marseille Université, 13015 Marseille, France; 6 Department of Radiology, La Conception Hospital, Assistance Publique-Hôpitaux de Marseille, Aix-Marseille University, 13005 Marseille, France; 7 Service de Chirurgie Thoracique Hopital Européen Georges Pompidou, Université Paris Cité, Paris, France; 8 AP-HP, Hopital Européen Georges Pompidou, Unité de Recherche Clinique, Centre d’Investigation Clinique 1418 Épidémiologie Clinique, INSERM, Université Paris Cité, Paris, France; 9 Service d’anatomie et Cytologie Pathologiques, Hôpital Nord, Chemin Des Bourrely, 13015 Marseille, France; 10 U1068-CRCM, Aix Marseille Université, 13015 Marseille, France; 11 Department of Radiology, AP-HM, Hôpital La Timone, 13005 Marseille, France; 12 Department of Neuroradiology, Alphonse de Rothschild Foundation Hospital, 75019 Paris, France; 13 AP-HP, Hopital Européen Georges Pompidou, PARCC UMRS 970, INSERM, Université Paris Cité, Paris, France

**Keywords:** Carcinoid tumors, Hamartomas, Pulmonary neoplasms, X-ray, Computed tomography, Radiomics

## Abstract

**Objectives:**

Lung carcinoids and atypical hamartomas may be difficult to differentiate but require different treatment. The aim was to differentiate these tumors using contrast-enhanced CT semantic and radiomics criteria.

**Methods:**

Between November 2009 and June 2020, consecutives patient operated for hamartomas or carcinoids with contrast-enhanced chest-CT were retrospectively reviewed. Semantic criteria were recorded and radiomics features were extracted from 3D segmentations using Pyradiomics. Reproducible and non-redundant radiomics features were used to training a random forest algorithm with cross-validation. A validation-set from another institution was used to evaluate of the radiomics signature, the 3D ‘median’ attenuation feature (3D-median) alone and the mean value from 2D-ROIs.

**Results:**

Seventy-three patients (median 58 years [43‒70]) were analyzed (16 hamartomas; 57 carcinoids). The radiomics signature predicted hamartomas vs carcinoids on the external dataset (22 hamartomas; 32 carcinoids) with an AUC = 0.76. The 3D-median was the most important in the model. Density thresholds < 10 HU to predict hamartoma and > 60 HU to predict carcinoids were chosen for their high specificity > 0.90. On the external dataset, sensitivity and specificity of the 3D-median and 2D-ROIs were, respectively, 0.23, 1.00 and 0.13, 1.00 < 10 HU; 0.63, 0.95 and 0.69, 0.91 > 60 HU. The 3D-median was more reproducible than 2D-ROIs (ICC = 0.97 95% CI [0.95‒0.99]; bias: 3 ± 7 HU limits of agreement (LoA) [− 10‒16] vs. ICC = 0.90 95% CI [0.85‒0.94]; bias: − 0.7 ± 21 HU LoA [− 4‒40], respectively).

**Conclusions:**

A radiomics signature can distinguish hamartomas from carcinoids with an AUC = 0.76. Median density < 10 HU and > 60 HU on 3D or 2D-ROIs may be useful in clinical practice to diagnose these tumors with confidence, but 3D is more reproducible.

**Critical relevance statement:**

Radiomic features help to identify the most discriminating imaging signs using random forest. ‘Median’ attenuation value (Hounsfield units), extracted from 3D-segmentations on contrast-enhanced chest-CTs, could distinguish carcinoids from atypical hamartomas (AUC = 0.85), was reproducible (ICC = 0.97), and generalized to an external dataset.

**Key points:**

• 3D-‘Median’ was the best feature to differentiate carcinoids from atypical hamartomas (AUC = 0.85).

• 3D-‘Median’ feature is reproducible (ICC = 0.97) and was generalized to an external dataset.

• Radiomics signature from 3D-segmentations differentiated carcinoids from atypical hamartomas with an AUC = 0.76.

• 2D-ROI value reached similar performance to 3D-‘median’ but was less reproducible (ICC = 0.90).

**Graphical Abstract:**

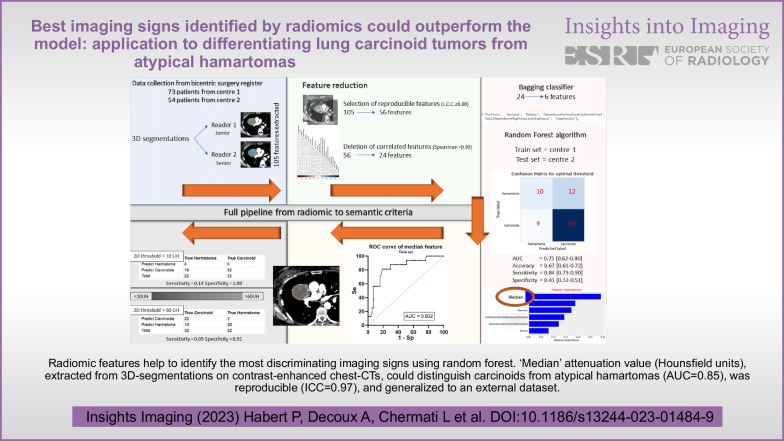

**Supplementary Information:**

The online version contains supplementary material available at 10.1186/s13244-023-01484-9.

## Introduction

Lung carcinoid tumors represent between 1 and 2% of primary pulmonary neoplasms in adults meaning 1.6 per 100,000 people in 2003 in the US [1, 2]. These tumors arise from neuroendocrine cells which are physiologically present throughout the lung tract [3]. The bronchopulmonary system is the second most frequent location of carcinoid tumors after the gastrointestinal tract [4]. Carcinoids have endobronchial localization in 85% of cases [2]. Typically, an atelectasis reveals the tumor with the macroscopic appearance of a “strawberry” when seen on bronchoscopy. Thanks to the wider use of chest CT, an increasing number of carcinoids are initially detected as incidental solid peripheral nodule [5]. In contrast, pulmonary hamartomas are benign tumors, often incidentally discovered and asymptomatic. Their histopathological characteristics may include fat, cartilage and epithelial tissue [6]. As the amount of fat and/or cartilage is variable [7], attenuation patterns on CT may vary and result in difficulties to non-invasively confirm the diagnosis. Based only on morphological CT features, hamartomas thus can mimic carcinoids, especially if there is neither macroscopic fat nor calcifications. Conversely, carcinoids may also contain fat and calcifications in varying proportions [8]. Clinically, both tumors are asymptomatic or present with cough or pneumonia if responsible for atelectasis [8, 9]. In these cases the radiological features of these tumors overlap, leading to difficulty in making a confident diagnosis by imaging and misdiagnosis is common. The differentiation between carcinoids and hamartomas is of clinical importance because localized forms of carcinoids require a surgical resection whereas no treatment is required for hamartomas. Surgery for these kinds of tumors vary from enucleation to pneumonectomy, with a morbidity rate reaching 15% for open thoracotomy [10]. There would be a clear clinical benefit to detect hamartomas pre-operatively, to avoid unnecessary surgery, without misdiagnosing a carcinoid tumor.

Radiomics is a data-driven research field using high-throughput mining of quantitative features extracted from medical images to discover new imaging biomarkers and enable phenotypic profiling of lesions [11]. There is an increasing interest for using radiomics to implement personalized medicine, especially in oncology [12] and in lung cancer [13]. As histological patterns for hamartomas and carcinoids are different, we hypothesized that radiomics may help differentiate these two tumor types.

The aim of this study therefore was to determine whether radiomics features on baseline lung contrast-enhanced CT images could distinguish carcinoids from atypical hamartomas.

## Materials and methods

**Fig. 1:**
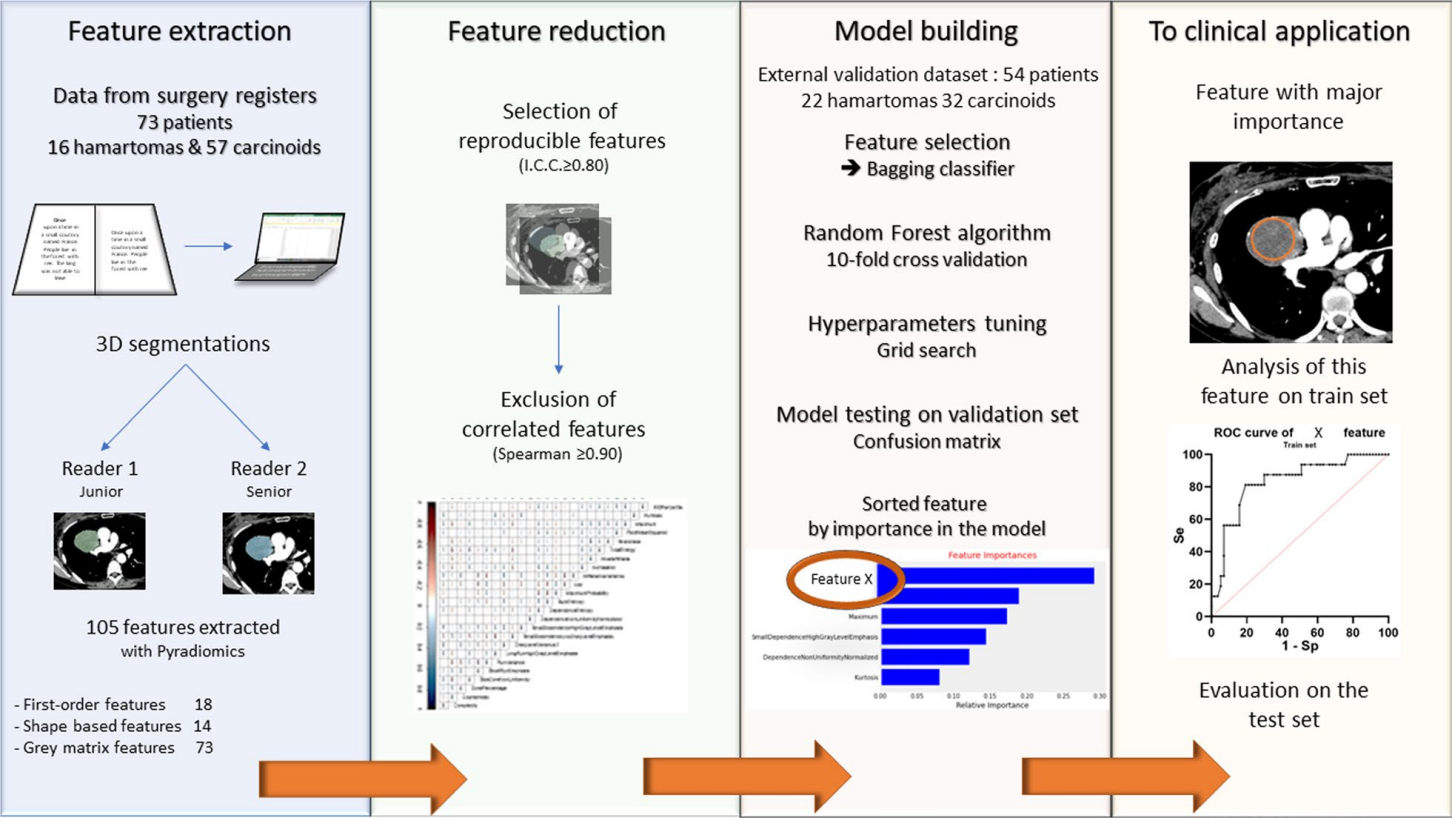
Illustrated full process of the study, from data curation to analyses

This retrospective study from two independent centers received approval from the Institutional Review Board (Comité d’Ethique pour la Recherche en Imagerie Médicale n°CRM-2202–229). One center was used as the training set to develop and cross-validate a model which was tested on an external validation set from the second institution (Fig. 1).

### Training set

Atypical hamartoma was defined as hamartomas misdiagnose in multidisciplinary tumor board and treated with surgery. Using a thoracic surgeons’ register, all patients with histologically-confirmed diagnosis of carcinoids or hamartomas after surgery between November 2009 and June 2020 from Hopital Nord – Marseille – APHM—France were collected. The inclusion criteria were: 1) a pre-operative chest CT available in the Picture Archiving and Communication System of the institutions, 2) presence of a post-contrast acquisition in a soft tissue reconstruction kernel, 3) slice thickness ≤ 2 mm. The exclusion criteria were: 1) two tumors in the same operated lobe, 2) tumor including less than 64 voxels as suggested in previous studies [14], and 3) insufficient image quality for the measures (motion or beam hardening artifacts).

Examinations came from different institutions and CT devices and therefore were performed with variable parameters (Supplementary Material). The median slice thickness was 1.25 mm [Q1–Q3, 1.25–1.50].

The age and gender of the patients, and the type of surgery performed were collected. Visual imaging signs on CT previously described for these tumors were analyzed: central or peripheral (i.e., sub-segmental bronchus or lower) localization; endobronchial position (if the tumor was located entirely or partially within bronchus); presence of atelectasis (partial or total); bronchial contact (distortion of a bronchus near the tumor); calcifications; border (lobulated or smooth). The longest diameter on the axial plane (mm) was also measured. These characteristics were recorded by a radiologist (L.C., 4 years of experience in chest imaging), blinded to histology.

### Feature extraction

**Fig. 2:**
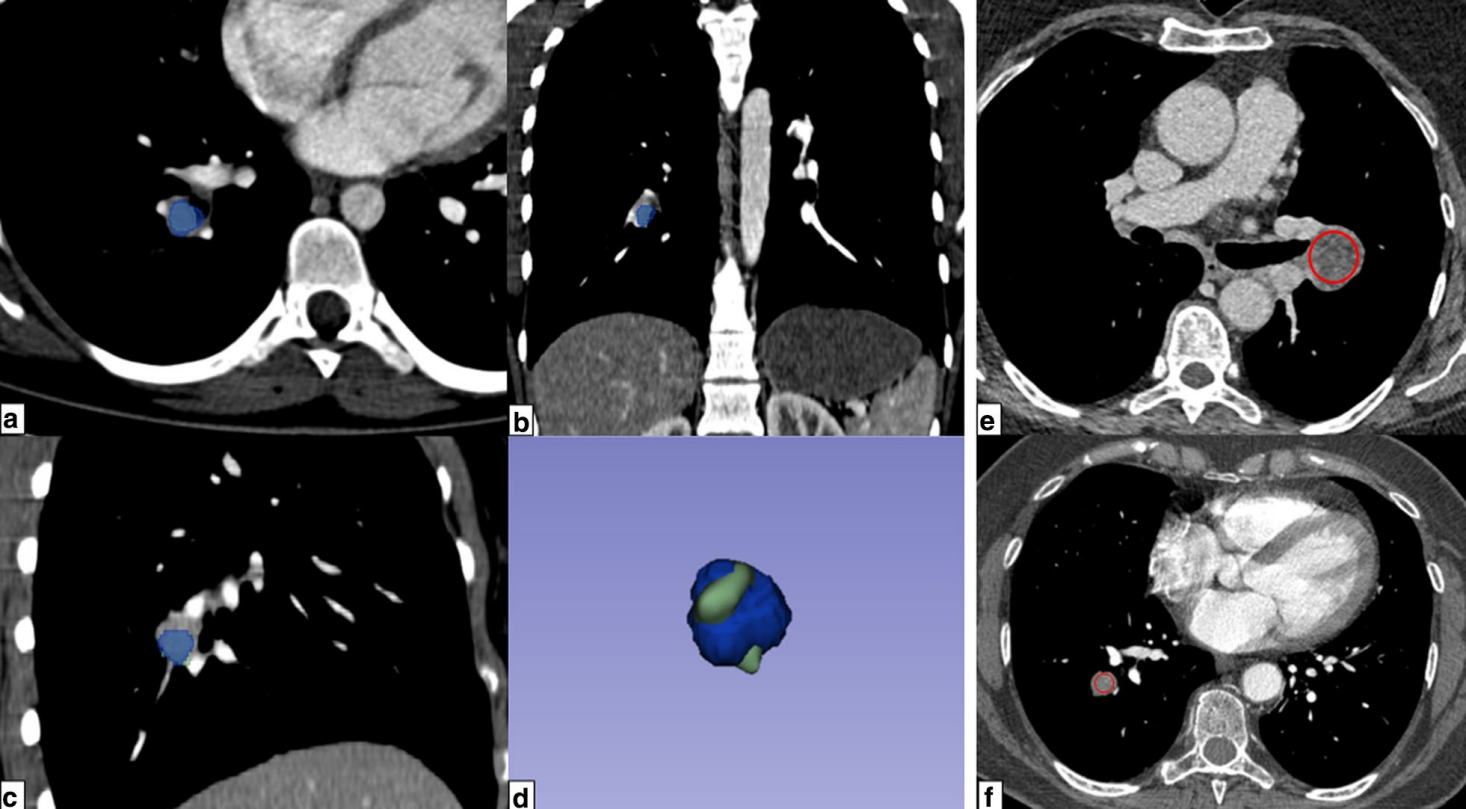
**a** axial, (**b**) coronal and (**c**) sagittal plane of contrast-enhanced chest CT in mediastinal window setting showing the 3D segmentation of one tumor, in blue, from which the ‘median’ feature was extracted (**d**) along with the volume rendering reconstruction of the merged segmentations of the two radiologists (blue and green) to illustrate reproducibility. **e**, **f** are two examples of red circular 2D ROIs used for measurement of mean attenuation. These circle was drawn using the dedicated tool of the viewer and place over 90% of the lesion, choosing the slice in which the tumor is the largest

Two radiologists (L.C. and P.H.) independently segmented volumes-of-interest (VOI) of the lesions on the soft kernel images, using 3D Slicer (version 4.7, National Institutes of Health–funded; https://www.slicer.org) [15, 16]. Large vessels and bronchi were not included. Radiologists were blinded to histology. The option to set the window width and window level was let to the radiologist preference within the software to efficiently delineate the nodule (examples in Fig. 2).

The Pyradiomics library (version 3.0.1, Computational Imaging & Bioinformatics Lab—Harvard Medical School) was used to extract radiomics features from the VOI using Python (version 3.8.8) [17]. Fixed bin width was set to 64 with no other preprocessing filter. One hundred five radiomics features were extracted, including shape-based (14 features), first-order statistics (18 features) and textural features (73 features). Their definitions are detailed in Additional file 1: Table e1 and follow the Image Biomarker Standardization Initiative guidelines [18].

**Table 1:** Population characteristics

	Hamartoma training set n = 16	Hamartoma validation set n = 22	*p*-value^$^	Carcinoid tumor training set n = 57	Carcinoid tumor validation set n = 32	*p*-value^§^
Age (years)	62 [43–76]	58 [50–64]	*p* = 0.68	58 [41–70]	62 [51–67]	*p* = 0.28
Gender						
Male	10 (62%)	11 (50%)	*p* = 0.53	10 (25%)	8 (25%)	*p* = 0.99
Female	6 (38%)	11 (50%)		47 (75%)	24 (75%)	
Type of surgery						
Pneumonectomy	0 (0%)	0 (0%)	*p* = 0.72	2 (3%)	2 (6%)	*p* = 0.94
Lobectomy	7 (44%)	1 (5%)		42 (74%)	26 (82%)	
Segmentectomy	0 (0%)	0 (0%)		8 (14%)	2 (6%)	
Wedge resection	7 (44%)	15 (68%)		5 (9%)	2 (6%)	
Enucleation	1 (6%)	6 (27%)		0 (0%)	0 (0%)	
Biopsy	1 (6%)	0 (0%)		0 (0%)	0 (0%)	
Diameter (mm)	17 [11–26]	12 [8–18]	*p* = 0.16	18 [12–26]	18 [13–25]	*p* = 0.95
Calcifications	7 (44%)	4 (18%)	*p* = 0.15	9 (16%)	2 (7%)	*p* = 0.32
Anatomic location						
Central	6 (37%)	4 (18%)	*p* = 0.27	30 (53%)	16 (50%)	*p* = 0.83
Peripheral	10 (62%)	18 (82%)		27 (47%)	16 (50%)	
Bronchial contact	5 (31%)	5 (23%)	*p* = 0.71	38 (67%)	25 (78%)	*p* = 0.33
Endobronchial protrusion	4 (25%)	0 (0%)	***p* = 0.02**	23 (40%)	16 (50%)	*p* = 0.50
Atelectasis	2 (13%)	0 (0%)	*p* = 0.17	16 (28%)	12 (38%)	*p* = 0.48
Borders shape						
Smooth	11 (69%)	11 (50%)	*p* = 0.33	40 (70%)	19 (61%)	*p* = 0.48
Lobulated	5 (31%)	11 (50%)		17 (30%)	12 (39%)	
Mean 2DROI value (HU)	30 [12–40]	26 [18–33]	*p* = 0.51	80 [50–103]	78 [57–107]	*p* = 0.99
Mean 2DROI value (HU) in pulmonary artery trunk	210 [144–283]	225 [186–336]	*p* = 0.35	229 [183–309]	179 [151–260]	***p* = 0.03**

Demographic and clinical data. Results were expressed in median [Q1-Q3] for quantitative data or number (percentage) for qualitative data. ‘n = ’ corresponds to the number of patients operated. HU—Hounsfield units. A *p*-value lower than 0.05 was considered as significant. ^$^ Comparison between hamartoma on training and validation set. ^§^ Comparison between carcinoids on training and validation set. *p*-value < 0.05 was highlighted in bold

### Feature reduction, selection, and model building

Feature reduction was performed based on inter-observer reproducibility and feature redundancy. Inter-observer reproducibility was evaluated for all features, and features presenting with pairwise intraclass correlation coefficients (ICC, two-way random effect, single rater, absolute agreement) < 0.8 were considered not reproducible [19] and excluded. Reproducible features were then compared two-by-two using a Spearman correlation, and highly correlated features with a coefficient ≥ 0.9 were considered redundant and only one was retained.

After feature reduction, a sequential step forward feature selection method was performed with a fivefold cross-validation setup to find the best performing feature combination on the training set. The scoring criterion was the area under the receiver operating characteristic curve (AUC). The radiomics signature that obtained the highest AUC value was selected to retraining a Random Forest Classifier (RF) with a tenfold cross-validation on the whole training set. The RF hyperparameters were fine-tuned using a grid search approach. The best operating point of the model was defined on the training set as the threshold maximizing the Youden index. AUC, sensitivity, and specificity were calculated. Their 95% CI were computed using bootstraps with 1000 repetitions.

### Analysis of performance of the most important feature

The radiomics signature features were analyzed according to their importance in the model. The most important feature was analyzed independently to determine whether it could predict histology on its own, using the senior radiologist’s segmentations. The threshold optimizing the Negative Likelihood Ratio for predicting hamartomas was determined. Two thresholds were chosen also to optimize respective specificity for each type of tumor.

To approximate the 3D feature using widely available clinical tools, mean attenuation was also calculated from a circle-shaped 2D-ROI placed over 90% of the lesion, on the slice in which the nodule was the largest, avoiding calcifications if present, and performed by the two radiologists.

### Reproducibility of 3D and 2D measures

The inter-reader reproducibility of the measure of the most important feature in the model (3D and 2D measure) was assessed on the training set using the ICC (two-way random effect, single rater, absolute agreement) and Bland–Altman method (bias, standard deviation of the bias, limits of agreement (LoA)). The reproducibility of three non-contiguous 2D measures within each tumor was assessed by the same methods.

### Correlation between contrast enhancement quality and the most important feature

To be sure that concentration of contrast did not influence the measure made on carcinoids, a Spearman correlation was performed between the aforementioned feature and mean attenuation value obtained with a 2D-ROIs drawn in the pulmonary artery trunk.

Carcinoids were split in two groups using the cut-off of 250 HU in the pulmonary artery trunk obtained with 2D-ROIs, as previously validated as a quality criterion for chest-CT arterial enhancement [20] and compared to ensure that contrast concentration did not influence the measure.

### External validation set

An external validation set from an independent center (Hopital Européen Georges Pompidou—APHP—Paris—France) was collected, identified from the pathology register, to test the model using the same inclusion and exclusion criteria as the training set. The lesions were delineated by one radiologist (P.H.), blinded to histology.

The radiomics signature, the three thresholds for the most important feature alone, and the 2D-derived measure were tested on this external validation dataset, and their performance was evaluated (AUC, sensitivity, specificity).

Corrected positive and negative predictive values (PPV and NPV) were calculated using the mean of the ten years’ prevalence observed in the two centers.

### Statistical analysis

Continuous data were expressed as median [Q1-Q3]. Categorical data were expressed as frequency or percentage. A two-sided *p*-value < 0.05 was considered statistically significant. The Radiomics Quality Score was calculated [18]. Quantitative data are given with their 95% CI. A Mann–Whitney test was used to compare quantitative data. To compare semantic criteria of hamartomas and carcinoid tumors Mann–Whitney test was used to compare quantitative data and Chi-square test to compare qualitative data.

The following packages of Python were used: Numpy (version 1.20.1) and Pandas (version 1.2.4) for data handling; Mlxtend (version 0.19.0) and Scikit-Learn (version 0.24.1) for preprocessing, machine learning, and performance evaluation; and matplotlib (version 3.3.4) for plots. The ICC function was assessed using R (version 3.6.1) from the IRR package (version 0.84.1).

## Results

### Training and external validation datasets

**Fig. 3:**
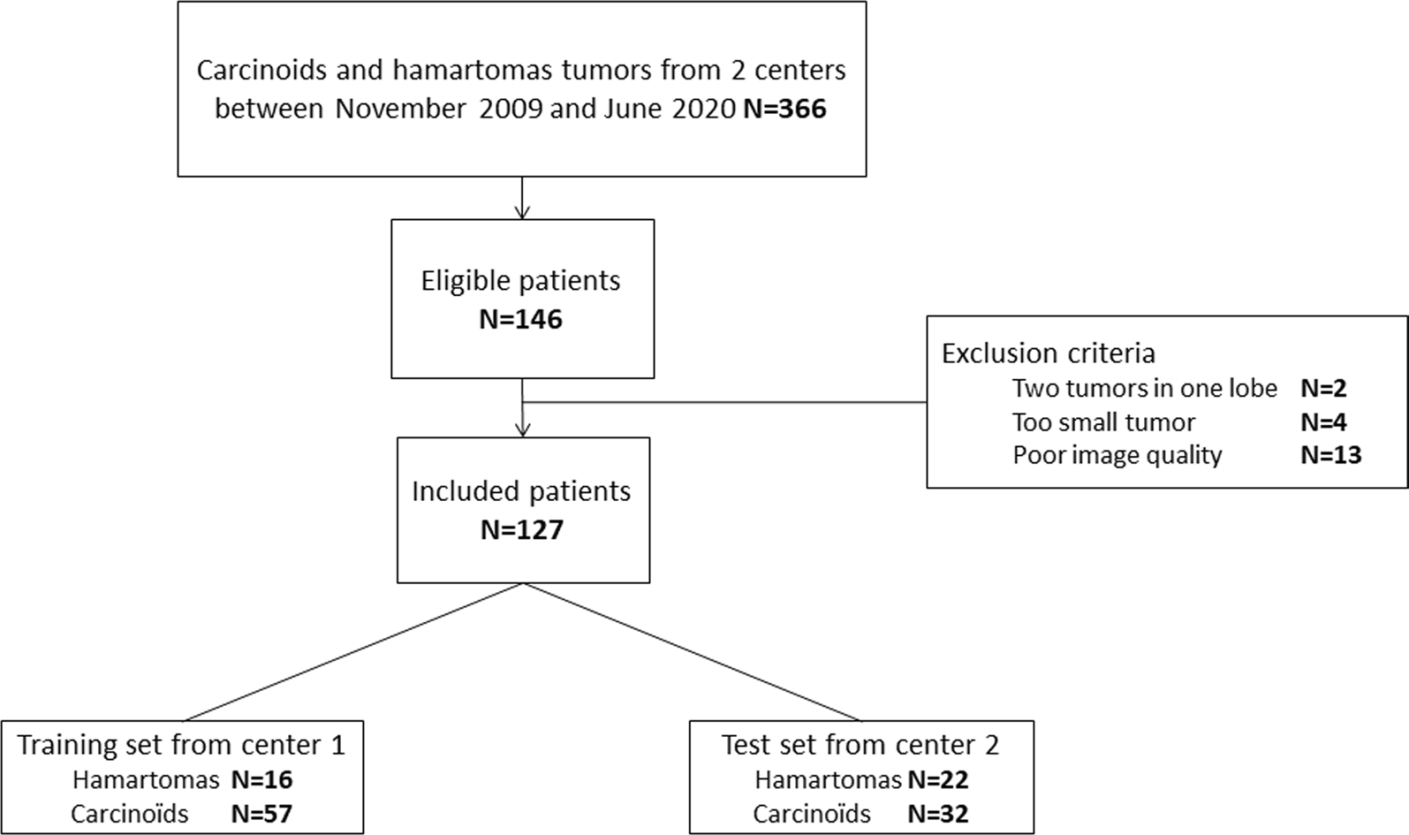
Flowchart of the study

Two hundred and six patients which had available post-operative histopathological reports of carcinoids or hamartomas were reviewed for the training set. Among them, 82 patients met the inclusion criteria. The following patients were excluded: two tumors in the same resected part of the lung (N = 1), small tumor size (N = 1), or insufficient image quality (N = 7). Finally, 73 patients with a median age of 58 [43–69] years, including 16 hamartomas and 57 carcinoids (42 typical and 15 atypical) were retrospectively analyzed (Fig. 3). The external validation dataset, following the same inclusion and exclusion criteria as the training set, included 54 patients (32 carcinoids including 25 typical and 7 atypical, and 22 hamartomas).

There was no statistically significant difference in semantic characteristics for each type of tumors between the training and external validation datasets, except for endobronchial protrusion in hamartomas which was more frequent in the training set (Table 1). Comparison between all hamartoma and carcinoid tumors has been added in supplemental material (Table 2).

**Table 2:** Comparison between hamartoma and carcinoid tumors

Population characteristics	Hamartoma n = 38	Carcinoid tumor n = 89	*p*-value
Age (years)	58 [49–67]	60 [45–68]	*p* = 0.93
Gender			
Male	21 (55%)	18 (20%)	***p* = 0.01**
Female	17 (45%)	71 (80%)	
Type of surgery			
Pneumonectomy	0 (0%)	4 (4%)	***p* = 0.01**
Lobectomy	8 (21%)	68 (76%)	
Segmentectomy	0 (0%)	10 (11%)	
Wedge resection	22 (58%)	7 (9%)	
Enucleation	7 (18%)	0 (0%)	
Biopsy	1 (3%)	0 (0%)	
Diameter (mm)	14 [10–24]	18 [12–26]	***p* = 0.03**
Calcifications	11 (44%)	11 (18%)	***p* = 0.02**
Anatomic location			
Central	10 (26%)	46 (52%)	***p* = 0.01**
Peripheral	28 (74%)	43 (48%)	
Bronchial contact	10 (26%)	63 (71%)	***p* = 0.01**
Endobronchial protrusion	4 (11%)	39 (44%)	***p* = 0.01**
Atelectasis	2 (5%)	28 (31%)	***p* = 0.01**
Borders shape			
Smooth	22 (58%)	59 (66%)	*p* = 0.37
Lobulated	16 (42%)	30 (34%)	
Mean 2DROI value (HU)	28 [17–36]	79 [55–105]	***p* = 0.01**

Demographic and clinical data. Results were expressed in median [Q1-Q3] for quantitative data or number (percentage) for qualitative data. ‘*n* = ’ corresponds to the number of patients operated. HU—Hounsfield units. A *p*-value lower than 0.05 was considered as significant. *p*-value < 0.05 was highlighted in bold

### Radiomics criteria

Median number of pixels in 3D VOIs was 2296 [Q1‒Q3, 417‒7856]. Median number of pixels in 2D ROIs was 200 [62‒428]. Radiomics feature reduction according to ICC ≥ 0.8 resulted in 56 reproducible features. Among them, 32 were redundant (Spearman correlation coefficient ≥ 0.9), leaving 24 features for subsequent analyses. The sequential step forward feature selection using the bagging classifier on the training set yielded a radiomics signature of five features that maximized the AUC value in distinguishing the two tumors (0.89 [95% CI: 0.81–0.98]).

**Fig. 4:**
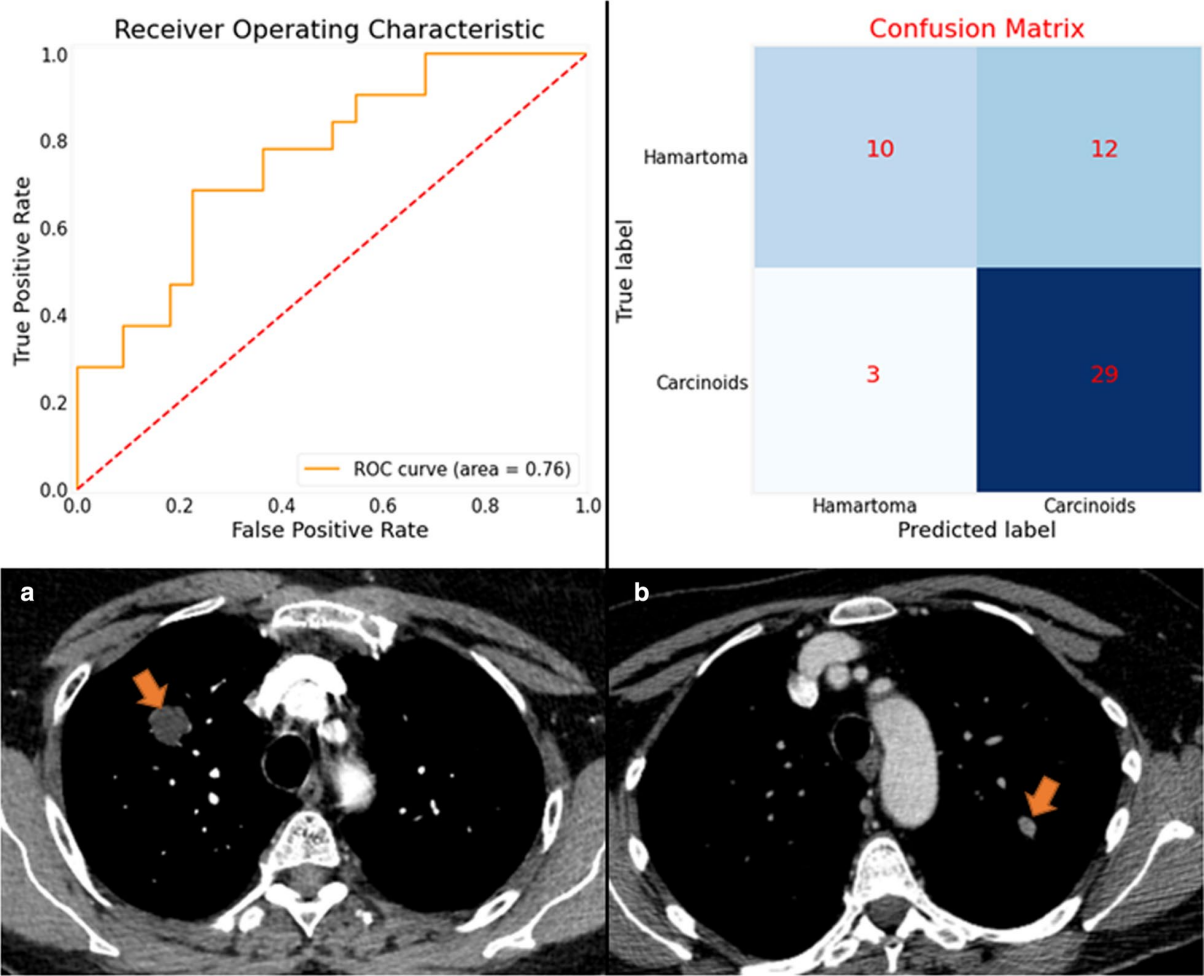
ROC curve of the RF model on the external validation set and the corresponding confusion matrix. Example of two axial slice of enhanced chest CTs showing a hamartoma (**a**) with a 3D median attenuation of − 15 HU and 2D mean attenuation of − 22 HU, and a carcinoid tumor (**b**) with a 3D median attenuation of 71 HU and a 2D mean attenuation of 77 HU

These features were: first-order features ('Median' and 'Maximum’ attenuation) and texture features ('DifferenceVariance,’ ‘SmallDependenceHighGrayLevelEmphasis' and 'Coarseness'). The Youden index was 0.61. When applied on the external validation set, the radiomics signature AUC, sensitivity and specificity were 0.76 [95% CI: 0.71–0.82], 91% [95% CI: 86–95%] and 46% [95% CI: 37–55%], respectively (Fig. 4). The importance of each feature in the model was 0.31, 0.26, 0.18, 0.15 and 0.10, respectively.

The Radiomics Quality Score was performed according to the standard for radiomics studies and was 47/100 (Additional file 1: Table e2).

### Most important feature 3D and 2D-ROIs thresholds

The radiomics 3D ‘median’ attenuation feature, corresponding to the median HU value in the 3D VOI, reached a cross-validated AUC score of 0.85 [95% CI: 0.74–0.96] on the training set (Additional file 1: Figure e1). We selected the following intensity thresholds to predict hamartoma or carcinoids with high specificity on the training set: < 10 HU to predict hamartomas (specificity 96%, [95% CI: 96–99%]), > 60 HU to predict carcinoids (specificity 68%, [95% CI: 55–79%]). The threshold that maximized the Negative Likelihood Ratio (4.9) was 40 HU, with a sensitivity of 69% ([95% CI: 44–86%]) and a specificity of 86% ([95% CI: 75–93%]) to predict hamartomas on the training set for tumors with a 3D ‘median’ attenuation value below 40 HU.

**Fig. 5:**
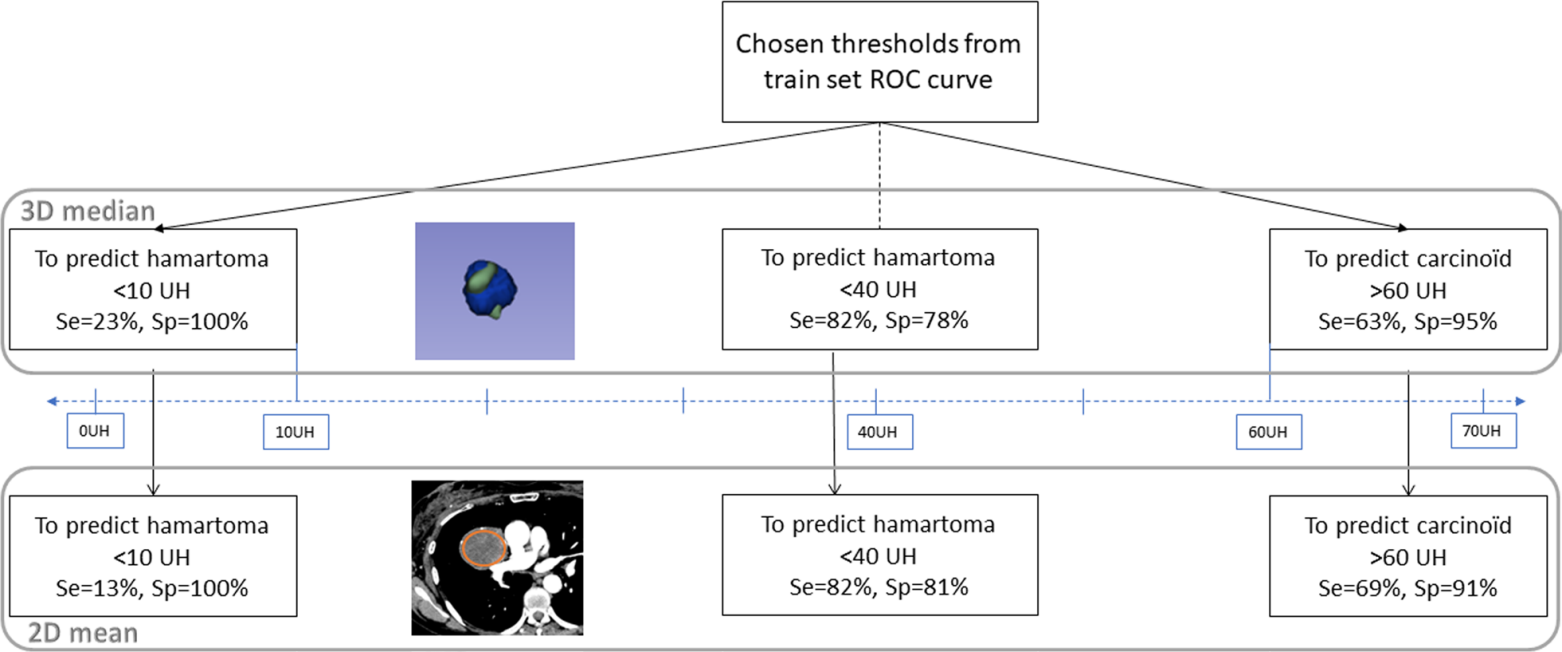
Results of the application of different thresholds selected from the training set ROC curve, to predict hamartoma of carcinoids on the external validation dataset using the ‘median’ feature extracted from 3D segmentations and the 2D mean attenuation

These thresholds were then applied on the external validation dataset using the 3D ‘median’ attenuation feature and the easy-to-use in clinical practice mean attenuation measured on the 2D-ROI. These results are summarized in Fig. 5 and detailed in Table 3. The positive and negative predictive values were calculated and corrected using the mean prevalence of hamartomas of the two centers (prevalence from the center one: 35%, from the center two: 18%, mean prevalence: 26%).

**Table 3:** Confusion matrix for different thresholds on validation set with corrected prevalence

To predict hamartoma	True hamartoma	True carcinoid	Sensitivity	Specificity	cPPV	cNPV
**3D < 10 HU**			0.23 [0.10–0.43]	1.00 [0.89–1.00]	1.00 [0.24–1.00]	0.79 [0.74–0.83]
Predict hamartoma	5	0				
Predict carcinoid	17	32				
**2D < 10 HU**			0.13 [0.05–0.33]	1.00 [0.89–1.00]	1.00 [0.14–1.00]	0.77 [0.73–0.81]
Predict hamartoma	3	0				
Predict carcinoid	19	32				
**Best threshold (To predict hamartoma)**	True hamartoma	True carcinoid	Sensitivity	Specificity	PPV	NPV
**3D < 40 HU**			0.82 [0.61–0.93]	0.78 [0.61–0.89]	0.57 [0.35–0.75]	0.93 [0.82–0.97]
Predict hamartoma	18	7				
Predict carcinoid	4	25				
**2D < 40 HU**			0.82 [0.61–0.93]	0.81 [0.65–0.91]	0.60 [0.34–0.78]	0.93 [0.83–0.97]
Predict hamartoma	18	6				
Predict carcinoid	4	26				
**To predict carcinoid**	True carcinoid	True hamartoma	Sensitivity	Specificity	PPV	NPV
**3D > 60 HU**			0.63 [0.45–0.77]	0.95 [0.78–0.99]	0.82 [0.42–0.96]	0.88 [0.80–0.92]
Predict carcinoid	20	1				
Predict hamartoma	12	21				
**2D > 60 HU**			0.69 [0.51–0.82]	0.91 [0.72–0.98]	0.73 [0.39–0.94]	0.89 [0.81–0.94]
Predict carcinoid	22	2				
Predict hamartoma	10	20				

This table illustrates different confusion matrix for different thresholds (< 10 and > 60 HU) chosen on the training set, measured using 2D or 3D segmentations on the external validation set. Best threshold was chosen according to highest Likelihood Ratio (= 4.9). The corrected prevalence was set to 26%. HU—Hounsfield units; cNPV—corrected negative predictive values; cPPV—corrected positive predictive values

### Reproducibility of the most important feature

**Fig. 6:**
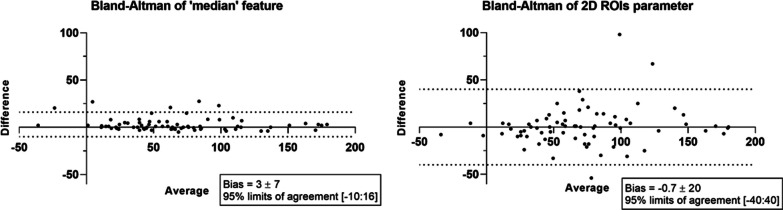
Bland and Altman plot, for difference in HU values measured between reader 1 and reader 2 with 3D ‘median’ attenuation feature on the left side and 2D mean attenuation on the right side. The ‘median’ feature is more reproducible than the 2D measure, with a smaller standard deviation of the bias which is more clinically relevant to the previously defined thresholds to diagnose carcinoids and atypical hamartomas

The ICC of the 3D ‘median’ attenuation feature and 2D mean attenuation were 0.97 ([95% CI: 0.95–0.99]) and 0.90 ([95% CI: 0.85–0.94]), respectively. The evaluation of reproducibility of the 3D ‘median’ attenuation feature and 2D-mean density using the Bland & Altman method showed that the 3D ‘median’ attenuation feature was more reproducible than the 2D mean attenuation (bias 3 ± 7 HU, LoA [–10–16] vs − 0.7 ± 20 HU, LoA [–40–40] (Fig. 6).

The ICC of mean values from 2D mean attenuation at three different levels of the tumor was excellent (0.93, [95% CI: 0.90–0.96]). But the LoA was wide and difficult to adapt to the scale previously described to predict hamartomas and carcinoids (Additional file 1: Figure e2).

There was no correlation between the pulmonary artery trunk enhancement and the 3D ‘median’ attenuation of carcinoids in the training set (r = 0.07), and no significant difference in the value of the 3D ‘median’ attenuation feature between chest CT with 2D-ROIs in pulmonary trunk > 250 HU versus those < 250 HU (*p* = 0.544) (Additional file 1: Figure e3).

## Discussion

Radiomics features allowed identifying imaging features differentiating lung atypical hamartomas from carcinoids, with an AUC of 0.76. The ‘median’ attenuation (HU) was the most important feature in the model, this feature alone and on the training set reached an AUC of 0.85. Best thresholds to predict hamartomas and carcinoids on the external dataset were < 10 HU and > 60 FHU, respectively. 2D mean attenuation measured on circular ROIs gave good results, with a difference in sensitivity and specificity below 10% compared to the 3D ‘median’ attenuation feature. The 3D ‘median’ attenuation feature was slightly more reproducible than the 2D mean attenuation. In this case the simple attenuation features outperform the model and was more efficient.

Typical hamartomas combining fat, tissue and cartilaginous calcifications are easy to diagnose but infrequent [21], especially when small. Hamartomas do not require treatment, but they can easily be confounded with carcinoid tumors when the radiological presentation is atypical that mean without calcification or fat, leading to unnecessary surgeries and potential complications. Clinical presentation could be the same: asymptomatic, cough, pneumonitis. Semantic radiological criteria have an area of overlap for atypical hamartomas and carcinoid tumors [22, 23]. To add difficulty, carcinoid tumors may present with calcifications, Huang et al. report a series on 21 cases [21], in which 12% of carcinoids contained calcifications while others studies found calcifications in 33% or 40% [23, 24]. Another area of overlap concerns the traditional “central and endobronchial” description of carcinoids (opposite to the description of “peripheral and extra bronchial” hamartoma) as it seems to have been overestimated in the past, as shown in recent studies (down from 84% in older series to 47–57% in recent series) [23–25] probably due to the increased use of CT. Presentation entirely inside the bronchial lumen is not the most frequent situation for carcinoids, 69% were not endobronchial in our study, similar to previous recent publications reporting 75% and 77% [23, 24]. As all these publications are retrospective and on small numbers of patients, sensitivity and specificity of semantic signs are not reported.

PET/CT may not have additional value as carcinoids and hamartomas both can present with either slightly increased or no ^18^FDG uptake [22]. The best tool seems ^68^ Ga-DOTATOC, achieving a detection rate of 88.4% with threshold of SUV_max_ > 2.5 but with a high rate of false-negatives [26].

Radiomics is a promising field for tumor characterization and has already proven its efficiency for carcinoids, to discriminate the different levels of Ki-67 expression or metastatic diseases [8]. From an initial strategy based on machine learning using radiomics features, we identified a five feature-signature with good results on an external validation dataset. We wished to explore whether this signature could be simplified. Using the 3D ‘median’ attenuation feature alone performed better on the external validation dataset than the RF algorithm and the signature. This illustrates the limitation of these signatures built on training sets, the meaning of which are often difficult to understand, and the challenge to find a generalizable, robust and reproducible signature for clinical practice [27]. There are different methods to reduce and select radiomics features. The extraction using the open-source Pyradiomics tool is today widely used thanks to its availability [28]. The chosen method to reduce features, removing non reproducible and redundant features, has already been published with good results [29] but there is not today a single accepted method.

Radiomics feature are dependent on acquisition parameters, such as pixel size and kernel [30]. Multiple CT machines were used in our study leading to heterogeneity in CT protocols. To limit this bias, we excluded patients with slice thickness > 2 mm, and we used only smooth kernel reconstructions for segmentations. The use of an external dataset to validate the model and the other diagnostic features is a key point in the IBSI guideline. Three-dimensional segmentations were drawn, more time consuming than 2D-segmentations, but gave results which seemed more reproducible for clinical use, and allowed getting rid of the inter-slice variability of 2D measure. The manual segmentations of tumors could introduce measurement bias, but we performed double independent segmentations and we controlled for reproducibility according to imaging biomarkers recommendations [31, 32].

This study has some limitations. We did not analyze all the hamartomas of the centers but only those who had been surgically removed. Though this led to a selection bias, this dataset represents hamartomas that are challenging in a clinical context, since they were not diagnosed pre-operatively. Finally, we tried to simplify the 3D measure by a 2D measure simpler to implement in routine, but calculated the mean instead of the median, as it was the measure most frequently available on clinical PACS. Mean value is influenced by extreme values while median is not, but due to the high number of pixels in each tumor, we hypothesized that the distribution of the HU value within tumors could tend toward a Gaussian distribution. However, though performance was similar for 2D and 3D, the latter’s higher reproducibility makes it a more reliable potential biomarker. We could guess what the goal of using radiomics in this study if simple attenuation feature outperforms the model. But to select these features and understand their importance in the machine learning model we must use radiomics. It seems that the second order features disturb the machine learning process and lower the performance of the model compared to the simple attenuation features.

In conclusion, a RF algorithm using radiomics features extracted from 3D-segmentations could differentiate atypical hamartomas from carcinoid tumors in lung on an external validation set with good performance (AUC = 0.76). Features based on HU participated for 57% in the model. The 3D ‘median’ attenuation alone reached an AUC = 0.85 on the training set. We propose diagnostic thresholds < 10 HU to confidently predict hamartomas and > 60 HU to confidently predict carcinoids with high specificity. 3D ‘median’ attenuation was a highly reproducible feature between two readers. The simpler 2D mean attenuation measurement was equally accurate but not reproducible enough between readers to be used.

### Supplementary Information

**Additional file 1:** Details of thoracic CT scans, radiomics signature, ROC curve of the "median" feature on the training set, legend of supplementary figures and RQS details.

## Data Availability

Data are available on reasonable request.

## Abbreviations

AUC: Area under curve
CT: Computed tomography
HU: Hounsfield units
LoA: Limits of agreement
NPV: Negative predictive value
PPV: Positive predictive value
RF: Random forest
ROC: Receive operating characteristics
