# Supplementary material for: Best imaging signs identified by radiomics could outperform the model: application to differentiating lung carcinoid tumors from atypical hamartomas
Source: Insights Imaging. 2023 Sep 19;14:148. doi: 10.1186/s13244-023-01484-9 (PMC10509085; doi:10.1186/s13244-023-01484-9)
Supplement: Supplementary file 1 — Additional file 1: Details of thoracic CT scans, radiomics signature, ROC curve of the "median" feature on the training set, legend of supplementary figures and RQS details. [file 13244_2023_1484_MOESM1_ESM.pdf]

Best imaging signs identified by radiomics could outperform the model: application to differentiating lung carcinoid tumors from atypical hamartomas

ELECTRONIC SUPPLEMENTARY MATERIAL

Chest CT-scanners details

In all cases, the matrix was 512x512 and the field of view 500.

Train set

Volumetric chest CT-scans of the lung were performed on 17 different 16 to 128 multi-slice CT models from 4 different manufacturers (Aquilion, Aquilion PRIME, Canon Medical System, Otawara, Japan ; BrightSpeed, Discovery CT750 HD, LightSpeed Pro 16, LightSpeed VCT, Optima CT 660, Revolution CT, Revolution EVO, Revolution HD, GE Healthcare, Milwaukee, WI, USA ; Brillance 64, Ingenuity CT, Philips Healthcare, Best, The Netherlands ; Emotion 16, Sensation 64, Sensation Cardiac 64, SOMATOM Definition, SOMATOM Definition AS+, SOMATOM Force, Siemens Healthcare, Erlangen, Germany). Acquisition parameters were not standardized, and tube voltage was set either to 100, 110, 120, 130 and 140 kV. Most of the examinations were acquired at 120kV (57/73; 78%). Median slice thickness was 1.25 mm [IQR = 0.25; range, 0.6 to 2 mm].

External validation set

CT-scans were performed on 15 different 16 to 128 multi-slice CT models from 4 different manufacturers (Aquilion, Aquilion PRIME, Canon Medical System, Otawara, Japan ; BrightSpeed, LightSpeed Pro 32, LightSpeed VCT, LightSpeed 16, Optima CT 660, GE Healthcare, Milwaukee, WI, USA ; Brilliance 64, Ingenuity Core, Philips Healthcare, Best, The Netherlands ; Perspective, Sensation 16, SOMATOM Definition AS+, SOMATOM Definition Edge, SOMATOM Force, SOMATOM Perspective, SOMATOM X.cite, Siemens Healthcare, Erlangen, Germany). Acquisition parameters were not standardized, and tube voltage was set either to 80, 90, 100, 110, 120, 130 and 140 kV. Most of the examinations were acquired at 120kV (32/54; 59%). Median slice thickness was 1.25 mm [IQR = 0.25; range, 0.6 to 2 mm].

Radiomic signature

first-order features: 'Median' and 'Maximum’; grey matrix features: 'DifferenceVariance’, ‘SmallDependenceHighGrayLevelEmphasis' and 'Coarseness'. The importance for each feature in the model was 0.31, 0.26, 0.18, 0.15 and 0.10 respectively.

Supplementary Figures

Figure e1: ROC curve from ‘median’ feature on the train set.

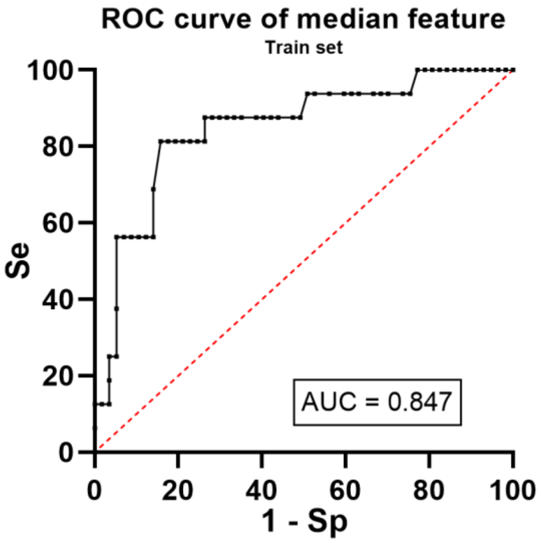

Figure e2: Reproducibility of 2D-ROIs parameter at different level of the tumors, up on the slice in which the nodule was the largest, and down. On the left-side of the panel an example of measure is illustrated with range calculated. On the right-side bar plot represent the distribution of maximum minus minimum UH value from the three 2D-ROIs.

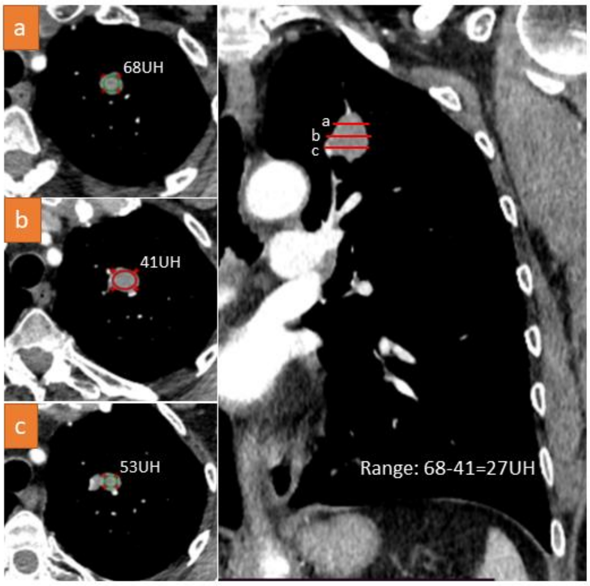

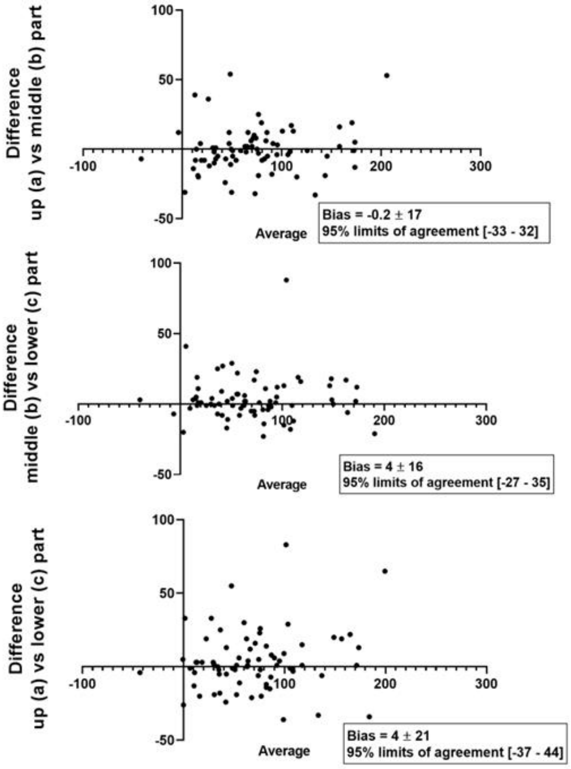

Figure e3: Correlation on the train set between 2D ROIs in pulmonary artery trunk and 'median' feature for carcinoids and difference of 'median' feature value for the threshold of 250 HU.

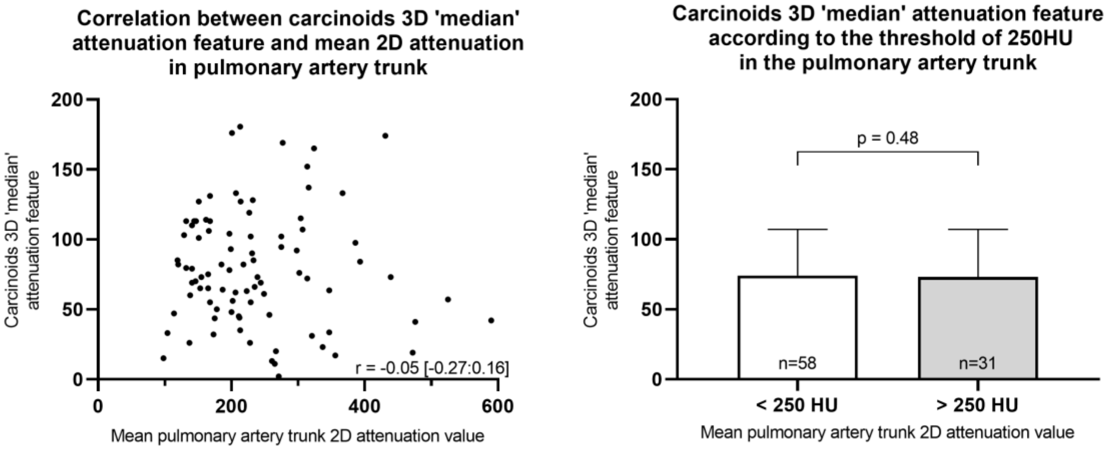

Supplementary Table

Table e1. Radiomic features extracted with PyRadiomics. Their mathematical definitions are available in the software documentation.

**Shape**	VoxelVolume,<br>Elongation,<br>SurfaceArea,<br>MeshVolume,<br>MinorAxisLength,<br>Maximum3DDiameter, Maximum2DDiameterSlice,<br>MajorAxisLength,<br>LeastAxisLength, Maximum2DDiameterColumn, Maximum2DDiameterRow,<br>Flatness,<br>SurfaceVolumeRatio,<br>Sphericity
**First-order**	Skewness,<br>90Percentile,<br>Maximum,<br>Median,<br>10Percentile,<br>Energy,<br>Range,<br>TotalEnergy,<br>Minimum,<br>Kurtosis,<br>RobustMeanAbsoluteDeviation,<br>Variance,<br>InterquartileRange,<br>Entropy,

	MeanAbsoluteDeviation,<br>Mean,<br>RootMeanSquared,<br>Uniformity
**Gray Level Dependence Matric (GLDM)**	SmallDependenceLowGrayLevelEmphasis,<br>DependenceEntropy,<br>LargeDependenceHighGrayLevelEmphasis,<br>LowGrayLevelEmphasis,<br>SmallDependenceEmphasis,<br>HighGrayLevelEmphasis,<br>GrayLevelVariance,<br>DependenceNonUniformityNormalized,<br>GrayLevelNonUniformity,<br>LargeDependenceEmphasis,<br>SmallDependenceHighGrayLevelEmphasis,<br>LargeDependenceLowGrayLevelEmphasis,<br>DependenceNonUniformity,<br>DependenceVariance
**Gray Level Co-occurrence Matric (GLCM)**	MaximumProbability,<br>ClusterProminence,<br>InverseVariance,<br>SumEntropy,<br>DifferenceEntropy,<br>Imc1,<br>ClusterShade,<br>Idn,<br>Autocorrelation,<br>Contrast,<br>JointEntropy,

	DifferenceVariance,<br><br>Idmn,<br><br>Idm,<br><br>Correlation,<br><br>Imc2,<br><br>JointAverage,<br><br>Id,<br><br>SumSquares,<br><br>ClusterTendency,<br><br>JointEnergy,<br><br>DifferenceAverage
**Gray Level Size Zone Matrix (GLSZM)**	LargeAreaLowGrayLevelEmphasis,<br><br>SmallAreaEmphasis,<br><br>SmallAreaHighGrayLevelEmphasis, LargeAreaHighGrayLevelEmphasis, SmallAreaLowGrayLevelEmphasis,<br><br>ZoneEntropy,<br><br>ZoneVariance,<br><br>SizeZoneNonUniformity,<br><br>LargeAreaEmphasis,<br><br>GrayLevelNonUniformity,<br><br>ZonePercentage,<br><br>LowGrayLevelZoneEmphasis,<br><br>GrayLevelVariance,<br><br>HighGrayLevelZoneEmphasis,<br><br>SizeZoneNonUniformityNormalized, GrayLevelNonUniformityNormalized
**Neighbouring Gray Tone Difference Matrix (NGTDM)**	Complexity,<br><br>Contrast,

	Strength,<br><br>Busyness,<br><br>Coarseness
**Gray Level Run Length Matrix (GLRLM)**	LongRunHighGrayLevelEmphasis,<br>ShortRunHighGrayLevelEmphasis,<br><br>RunLengthNonUniformity,<br><br>LongRunLowGrayLevelEmphasis,<br><br>LongRunEmphasis,<br><br>GrayLevelVariance,<br><br>RunVariance,<br><br>LowGrayLevelRunEmphasis,<br><br>GrayLevelNonUniformity,<br><br>ShortRunEmphasis,<br><br>ShortRunLowGrayLevelEmphasis,<br><br>RunEntropy,<br><br>RunLengthNonUniformityNormalized,<br><br>RunPercentage,<br><br>GrayLevelNonUniformityNormalized,<br><br>HighGrayLevelRunEmphasis

Table e2 Radiomic Quality Score

1	Image protocol quality	+ 1 protocols are well-documented
2	Multiple segmentations - Analyze feature robustness to segmentation variabilities	+ 1 multiple segmentations were performed
3	Phantom study on all scanners	+ 0
4	Imaging at multiple time points	+ 0
5	Feature reduction or adjustment for multiple testing	+ 3 feature reduction was implemented
6	Multivariable analysis with non-radiomics features (for example, EGFR mutation)	+ 1 multivariable with semantic criteria
7	Detect and discuss biological correlates	+ 1 discussed with histopathological correlates
8	Cut-off analyses	+ 1 cut-off analyses were provided
9	Discrimination statistics - One can also apply resampling method (for example, bootstrapping, cross-validation)	+ 1 discrimination statistic and its statistical significance are reported<br>+ 1 a resampling method technique is also applied
10	Calibration statistics - report calibration statistics. One can also apply resampling method.	+ 1 a calibration statistic and its statistical significance are reported + 1 a resampling method technique is also applied
11	Prospective study registered in a trial database	+ 0
12	Validation	+ 3 validation is based on a dataset from another institute
13	Comparison to ‘gold standard’	+ 0
14	Potential clinical utility	+ 2 potential clinical utility for diagnosis
15	Cost-effectiveness analysis	+ 0
16	Open science and data	+ 0
Total points (17/36 = 47%)		
